# B cell peripheral tolerance is promoted by cathepsin B protease

**DOI:** 10.1073/pnas.2300099120

**Published:** 2023-04-11

**Authors:** Marissa Y. Chou, Dan Liu, Jinping An, Ying Xu, Jason G. Cyster

**Affiliations:** ^a^Department of Microbiology and Immunology, University of California, San Francisco, CA 94143; ^b^HHMI, University of California, San Francisco, CA 94143

**Keywords:** B lymphocyte, signal-1, self-tolerance, cathepsin, CD40

## Abstract

B cells that bind soluble autoantigens receive chronic signaling via the B cell receptor (signal-1) in the absence of strong costimulatory signals (signal-2), and this leads to their elimination in peripheral tissues. The factors determining the extent of soluble autoantigen-binding B cell elimination are not fully understood. Here we demonstrate that the elimination of B cells chronically exposed to signal-1 is promoted by cathepsin B (Ctsb). Using hen egg lysozyme-specific (HEL-specific) immunoglobulin transgenic (MD4) B cells and mice harboring circulating HEL, we found improved survival and increased proliferation of HEL-binding B cells in Ctsb-deficient mice. Bone marrow chimera experiments established that both hematopoietic and nonhematopoietic sources of Ctsb were sufficient to promote peripheral B cell deletion. The depletion of CD4^+^ T cells overcame the survival and growth advantage provided by Ctsb deficiency, as did blocking CD40L or removing CD40 from the chronically antigen-engaged B cells. Thus, we suggest that Ctsb acts extracellularly to reduce soluble autoantigen-binding B cell survival and that its actions restrain CD40L-dependent pro-survival effects. These findings identify a role for cell-extrinsic protease activity in establishing a peripheral self-tolerance checkpoint.

## Significance

Self-tolerance in the B cell compartment of soluble autoantigens depends on mechanisms that promote peripheral B cell elimination. In this study, we report that deficiency in the cysteine protease cathepsin B (Ctsb) in mice results in less efficient removal of chronically antigen-engaged B cells from the repertoire. The tolerogenic action of Ctsb is lost in mice lacking the CD40 pathway, and the findings suggest that Ctsb restrains, directly or indirectly, basal CD40L-CD40 pathway activity and thereby helps prevent the activation of autoreactive B cells. Since protease activity is increased at sites of inflammation, our findings point to the possibility that self-tolerance checkpoints are reset in these sites due to proteolytic modulation of cell surface interaction molecules.

Self-t.olerance in the B cell compartment is established through multiple checkpoints (1). Antigens that strongly cross-link the B cell receptor (BCR) on developing B cells in the bone marrow (BM) cause receptor editing or deletion. B cells that recognize low-valency soluble autoantigens are not held up at the immature B cell checkpoint but are instead regulated by peripheral checkpoints. One model that has been widely used to study tolerance induction in response to soluble autoantigen involves immunoglobulin (Ig) transgenic mice (called MD4) that are specific for hen egg lysozyme (HEL) and transgenic mice that express soluble HEL as a neoself-antigen (called ML5) (2). In double-transgenic mice generated by intercrossing these two lines, the B cells are all autoantigen-engaged and thus chronically receiving B cell receptor (BCR) signals (signal-1). These HEL-binding B cells are not receiving cognate T cell help or being exposed to pathogen-associated molecular patterns (PAMPS) and thus are not receiving signal-2. HEL-engaged B cells have reduced surface IgM levels and a reduced ability to signal in vitro in response to exogenous HEL. When these chronically HEL-engaged B cells are placed in the polyclonal repertoire of wild-type (WT) mice, they undergo follicular exclusion and are deleted from the periphery within a few days (3, 4). This competitive elimination of B cells receiving chronic signal-1 without signal-2 occurs in part due to an increased dependence of the cells on the pro-survival factor BAFF and the limited availability of this factor in mice with a polyclonal B cell repertoire (5, 6). As well as being strongly dependent on BAFF, survival of B cells receiving chronic signal-1 in the absence of signal-2 is augmented by naïve CD4^+^ T cells (7). Although naïve CD4^+^ T cells are not conventionally considered to be a source of CD40L (and thus signal-2), our previous work showed that naïve CD4^+^ T cells constitutively express CD40L, though it is not measurable on the cell surface due to continual modulation by engagement with CD40-expressing cells; when these cells are removed, surface CD40L can be detected (8). Whether additional extrinsic factors beyond BAFF and naïve CD4^+^ T cell CD40L influence the survival of peripheral B cells chronically exposed to signal-1 is unclear.

Cathepsin B (Ctsb) is a widely expressed member of the cysteine cathepsin family (9). It is expressed as a preproenzyme in the endoplasmic reticulum, and it becomes a mature protease in the lysosome. It carries out a variety of functions in the lysosome, such as processing other lysosomal enzymes and mediating the degradation of hormones (9, 10). Ctsb is also found in multiple extracellular locations. Extracellular functions attributed to Ctsb include proteolysis of the extracellular matrix (11), protection of cytotoxic T cells from self-killing (12), generation of soluble TRAIL (TNFSF10) (13), activation of TGFb (14), and cleavage of several chemokines (15). In some of these studies, the extracellular function was thought to occur while Ctsb was associated with the surface of the secreting cell (10). Of particular relevance to B cell biology, a recent study suggested that extracellular Ctsb was required for cleavage of CXCL13 to help establish a gradient of this chemokine for the organization of B cell follicles in lymphoid tissues (16).

In this project, we set out to further characterize the influence of Ctsb on B cell follicle organization and function. In the Ctsb-deficient mice studied here, we did not observe a defect in follicular organization. However, when MD4 B cells were transferred into Ctsb-deficient mice containing soluble HEL such that they experienced chronic signal-1, the extent of B cell deletion was reduced compared to transfers into matched WT mice. Our experiments establish that hematopoietic and nonhematopoietic cells both contribute to the production of Ctsb that promotes HEL-binding B cell elimination. The effect of Ctsb on HEL-binding B cells was overcome in mice depleted of CD4^+^ T cells or deficient in CD40L function or in CD40. These findings suggest a pathway by which extracellular protease activity promotes B cell self-tolerance.

## Results

### Ctsb-Deficient Mice Have Normal Follicles but Reduced Elimination of HEL-Binding B Cells.

**Fig. 1.**
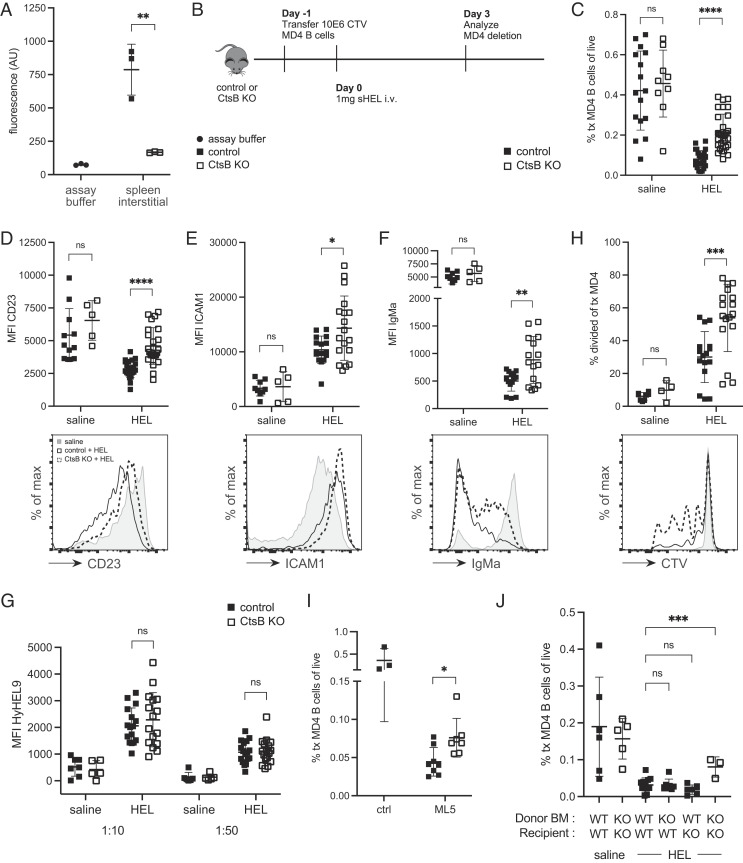
Ctsb Promotes Elimination of HEL-Binding B Cells. (*A*) Ctsb activity in assay buffer or spleen interstitial fluid of control (Ctsb^+/+^ or Ctsb^+/−^) or Ctsb^−/−^ (CtsB KO) mice. Data show results from two separate experiments. (*B*) Schematic of CTV-labeled MD4 B cell adoptive transfer into control or Ctsb-deficient mice, followed by HEL treatment. (*C*) Frequencies of transferred MD4 B cells in spleens of control or Ctsb-deficient recipients 3 d after saline (n = 16 control, n = 9 KO mice) or HEL treatment (n = 26 control, n = 30 KO mice). (*D*–*F*) MFI (*Top*) and representative histogram plot (*Bottom*) of CD23 (*D*), ICAM1 (*E*), and IgMa (*F*) on transferred MD4 B cells 3 d after saline (n = 11 control, n = 5 KO) or HEL treatment (n = 22 control, n = 24 KO). (*G*) MFI of HyHEL9 on MD4 B cells incubated with 1:10 or 1:50 dilutions of sera from control or Ctsb-deficient mice 3 d after saline (n = 7 control, n = 5 KO) or HEL treatment (n = 16 control, n = 17 KO). (*H*) Percentage of divided transferred MD4 B cells (*Top*) or representative histogram plot of CTV (*Bottom*) 3 d after saline (n = 8 control, n = 4 KO) or HEL treatment (n = 16 control, n = 17 KO). (*I*) Frequencies of transferred MD4 B cells in spleens of control ML5^+^ (n = 8) or Ctsb-deficient ML5^+^ (n = 7) recipients 3 d after MD4 B cell adoptive transfer. Control ML5^−^ (n = 3) mice used as deletion control. (*J*) Frequencies of transferred MD4 B cells in spleens of bone marrow (BM) chimeras 3 days after saline or HEL treatment. The Ctsb genotype of donor BM and recipient mice used to generate the BM chimeras is indicated. In graphs, each data point indicates an individual mouse and lines indicate means. Error bars represent SDs. *I* is representative of three experiments. Statistical significance for *A*–*I* was determined by unpaired *t* test. NS, not significant; *P < 0.05; **P < 0.01; ***P < 0.001; ****P < 0.0001. Statistical significance for *J* was determined by ordinary one-way ANOVA, ***, *P* = 0.0007.

Follicular organization in Ctsb-deficient mice was examined by staining spleen and lymph node sections for B cell and follicular dendritic cell (FDC) markers. Since the prior study had reported a requirement for Ctsb to maintain primary follicles and FDCs (16), we examined unimmunized mice. In the Ctsb^−/−^ mouse colony studied here, lymphoid follicle organization in spleen and lymph nodes was intact and FDCs were readily detected (*SI Appendix*, Fig. S1 *A* and *B*). Analysis of Ctsb activity in spleen interstitial fluid using an enzyme activity assay confirmed the Ctsb deficiency (Fig. 1*A*). These data suggest Ctsb is not essential for establishing the CXCL13 activity needed for follicular organization.

We next considered whether Ctsb might influence the fate of different types of follicular B cells. Ctsb-deficient mice had normal frequencies of splenic B cells and CD4^+^ T cells and slightly reduced CD8^+^ T cells (*SI Appendix*, Fig. S1*C*) as previously reported (16). The mice mounted intact splenic germinal center (GC) responses to sheep red blood cells (SRBCs), a model T-dependent antigen (*SI Appendix*, Fig. S1*D*). The chronic GC responses present in mesenteric lymph nodes and Peyer’s patches were also comparable between Ctsb-deficient and control mice (*SI Appendix*, Fig. S1*E*). Since the naïve and GC B cell compartments appeared intact, we next asked whether the fate of B cells experiencing chronic signal-1 was affected. This was tested in Ctsb-deficient mice by taking advantage of the finding that when mice harboring low frequencies of MD4 B cells are injected with large amounts of soluble HEL, the HEL-binding B cells experience chronic BCR engagement and are deleted within 3 d (7). This outcome is analogous to the loss that occurs when MD4 B cells are transferred into ML5 mice that express HEL endogenously (4). Using this MD4 cell transfer and HEL injection approach, we observed the expected marked elimination of splenic HEL-binding B cells in control recipients (Fig. 1 *B* and *C*). However, the extent of deletion was significantly reduced in Ctsb-deficient mice (Fig. 1*C*). Similar findings were made in peripheral lymph nodes (*SI Appendix*, Fig. S2*A*). Analysis of the surface phenotype of the transferred cells showed higher CD23 and ICAM1 levels on HEL-binding B cells in the Ctsb-deficient recipients (Fig. 1 *D* and *E*). The reduced expression of CD23 on HEL-engaged B cells is consistent with the ability of BCR signaling to downmodulate this marker (17). Surface IgM was also present at higher levels on a fraction of the HEL-binding B cells in Ctsb-deficient recipients (Fig. 1*F*). Since IgM downmodulation is highly sensitive to the extent of HEL exposure, we examined serum HEL abundance in the treated control and Ctsb-deficient mice. This was done by incubating MD4 B cells with serum taken from control and Ctsb-deficient mice at day 3 after HEL injection and then staining with HyHEL9 to detect the amount of bound HEL. Control and Ctsb-deficient mouse serum contained comparable amounts of HEL (Fig. 1*G*), thereby excluding differences in HEL availability as an explanation for the improved survival of HEL-binding B cells in mice lacking Ctsb. Using cell trace violet (CTV) labeling to track cell division in the transferred cells, few cells had divided in control recipients as expected, but there was an increase in cell division in Ctsb-deficient recipients (Fig. 1*H*). The assessment of the frequencies of divided and undivided B cells showed that both were increased (Fig. 1*H* and *SI Appendix*, Fig. S2*B*), indicating that Ctsb-deficiency augmented both proliferation and survival of B cells experiencing chronic BCR signaling. Analysis of the surface marker phenotype in divided and undivided transferred B cells showed a greater difference among the divided B cells, suggesting the mechanism promoting proliferation also contributed to the increased CD23, ICAM1, and IgM (*SI Appendix*, Fig. S2 *C*–*E*).

To confirm that the HEL injection approach was an accurate model for events occurring during autoantigen exposure, we crossed Ctsb^−/−^ mice with ML5 mice. Transferred MD4 cells were mostly deleted within 3 days of transfer into control ML5 mice, but the deletion was reduced in Ctsb^−/−^ ML5 mice (Fig. 1*I*). Serum HEL concentrations in ML5 mice were unchanged by Ctsb deficiency (*SI Appendix*, Fig. S2*F*). The HEL-binding B cells in Ctsb-deficient ML5 mice showed elevated CD23, ICAM1, and IgM (*SI Appendix*, Fig. S2 *G*–*I*), and the fraction of cells that divided was also increased (*SI Appendix*, Fig. S2*J*). Thus, Ctsb contributes to restraining HEL-autoantigen binding B cell activation and to promoting their removal from the peripheral B cell repertoire.

To test for possible effects of Ctsb on BAFF abundance, we took advantage of the finding that the BAFF availability is reflected in the amount that is bound to receptors on the B cell surface (5). Using a polyclonal anti-BAFF serum, the amount of surface BAFF on B cells from control and Cstb-deficient mice was equivalent (*SI Appendix*, Fig. S3*A*). As the size of the B cell compartment is highly responsive to BAFF availability (18), the unchanged size of the B cell compartment in Ctsb-deficient mice (*SI Appendix*, Fig. S1*C*) provided further evidence that BAFF abundance was not altered by Ctsb deficiency.

### Ctsb from Hematopoietic and Nonhematopoietic Cells Promotes Peripheral B Cell Tolerance.

Our transfer studies utilized WT MD4 B cells and thus established that the action of Ctsb in promoting elimination of B cells receiving chronic signal-1 was cell-extrinsic. To determine whether Ctsb was needed in hematopoietic or nonhematopoietic cells, we generated WT -> Ctsb KO, Ctsb KO -> WT, and Ctsb KO -> Ctsb KO BM chimeras. After 7 to 8 wk of reconstitution, MD4 B cells were transferred, and soluble HEL injected. The deletion efficiency was intact in all the chimeras except the Ctsb KO -> Ctsb KO group (Fig. 1*J*). These data indicate that Ctsb from either hematopoietic cells or radiation-resistant (most likely nonhematopoietic stromal) cells was sufficient for promoting elimination of HEL-binding B cells.

### Follicular Exclusion Is Intact in the Absence of Ctsb.

**Fig. 2.**
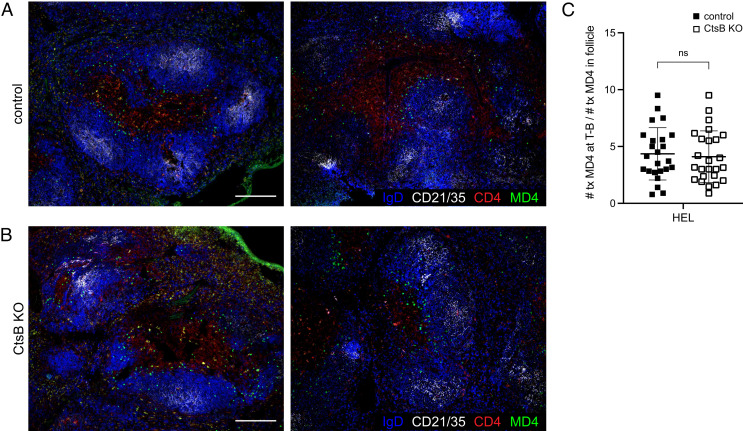
Follicular Exclusion of HEL-Engaged B Cells Is Unaffected by Ctsb Deficiency. (*A* and *B*) Immunofluorescence for MD4 GFP B cells (GFP, green) transferred into HEL-treated control (*A*) or Ctsb-deficient (*B*) mice stained to detect endogenous B cell follicles (IgD, blue; CD21/35, white) and the T cell zone (CD4, red). Sections were prepared one day after HEL treatment. Two example images are shown and are representative of multiple cross-sections from at least three mice of each type. (Scale bar, 200 µm.) (*C*) Quantification of proportion of MD4 GFP B cells at the T zone–follicle (T-B) interface one day after HEL treatment. Each data point represents an individual follicle (n = 24 control, n = 25 KO) from sections prepared from at least three mice of each genotype. Lines indicate means, and error bars represent SDs. Statistical significance was determined by unpaired *t* test. NS, not significant.

To test whether follicular exclusion of HEL-binding B cells was impaired in the absence of Ctsb, spleens were taken from control and Ctsb-deficient mice that harbored MD4 B cells and had been treated with soluble HEL 1 d earlier. Imaging analysis revealed the MD4 B cells were predominantly located at the follicle T zone interface in both control and Ctsb-deficient recipients (Fig. 2 *A*–*C*). Thus, Ctsb is not required for follicular exclusion of antigen-engaged B cells.

### CD4^+^ T Cell Depletion Overcomes the Effect of Ctsb Deficiency on HEL-Binding B Cells.

**Fig. 3.**
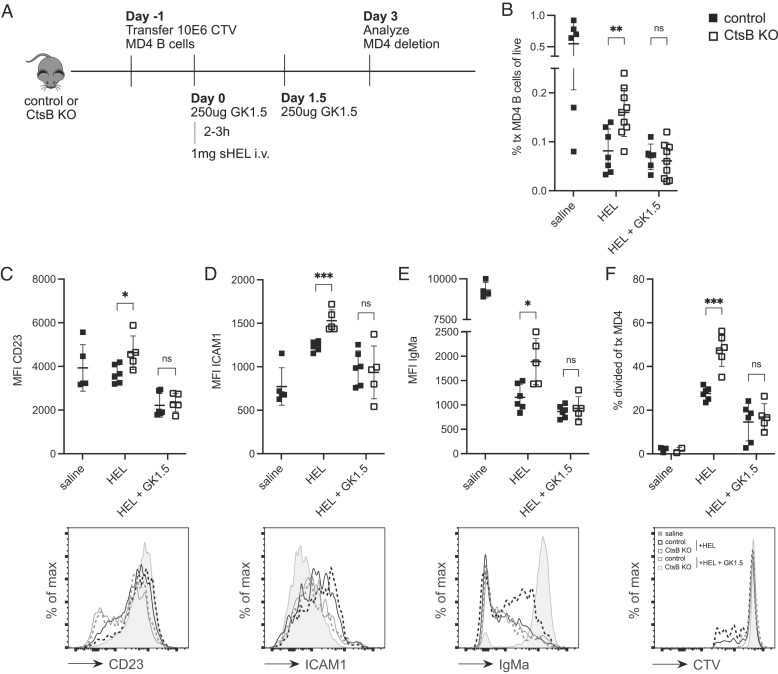
Depletion of CD4^+^ T Cells Overcomes the Effect of Ctsb Deficiency on HEL-Binding B Cells. (*A*) Schematic of CTV-labeled MD4 B cell adoptive transfer into control or Ctsb-deficient mice, followed by GK1.5 CD4^+^ T cell depletion and HEL treatment. (*B*) Frequencies of transferred MD4 B cells in spleens of control or Ctsb-deficient recipients 3 d after saline (n = 6 control), HEL (n = 7 control, n = 9 KO), or HEL with GK1.5 treatment (n = 6 control, n = 9 KO). (*C*–*E*) MFI (*Top*) and representative histogram plot (*Bottom*) of CD23 (C), ICAM1 (*D*), and IgMa (*E*) on transferred MD4 B cells 3 d after saline (n = 5 control), HEL (n = 6 control, n = 5 KO), or HEL with GK1.5 treatment (n = 6 control, n = 5 KO). (*F*) Percentage of divided transferred MD4 B cells (*Top*) or representative histogram plot of CTV (bottom) 3 d after saline (n = 3 control, n = 2 KO), HEL (n = 6 control, n = 6 KO), or HEL with GK1.5 treatment (n = 6 control, n = 5 KO). Each data point indicates an individual mouse and lines indicate means. Error bars represent SDs. *C*–*F* are representative of three experiments. Statistical significance for *B*–*F* was determined by unpaired *t* test. NS, not significant; *P < 0.05; **P < 0.01, ***P < 0.001.

Since prior work had established that naïve CD4^+^ T cells restrain the extent of HEL-binding B cell elimination in WT mice (7), we tested whether the action of Ctsb was influenced by CD4^+^ T cells. Control and Ctsb-deficient mice were treated with a CD4^+^ T cell depleting antibody and then used as recipients of MD4 cells and soluble HEL (Fig. 3*A*). In contrast to the findings in non-treated recipients, there was a similar extent of deletion in control and Ctsb-deficient mice when CD4^+^ T cells were lacking (Fig. 3*B*). There was also a loss in the increased levels of CD23, ICAM1 and IgM (Fig. 3 *C*–*E*). The effect of Ctsb-deficiency on HEL-binding B cell proliferation was also lost in CD4^+^ T cell-depleted mice (Fig. 3*F*). These data suggest that the B cell deletion-promoting effect of Ctsb may involve restraining some function of CD4^+^ T cells. Previous work had found that naïve CD4^+^ T cells can limit the extent of HEL autoantigen-binding B cell elimination through provision of CD40L (8). Therefore, we next examined the effect of CD40L blockade.

### Enhanced HEL-Binding B Cell Persistence in Ctsb-Deficient Hosts Depends on CD40L and CD40.

**Fig. 4.**
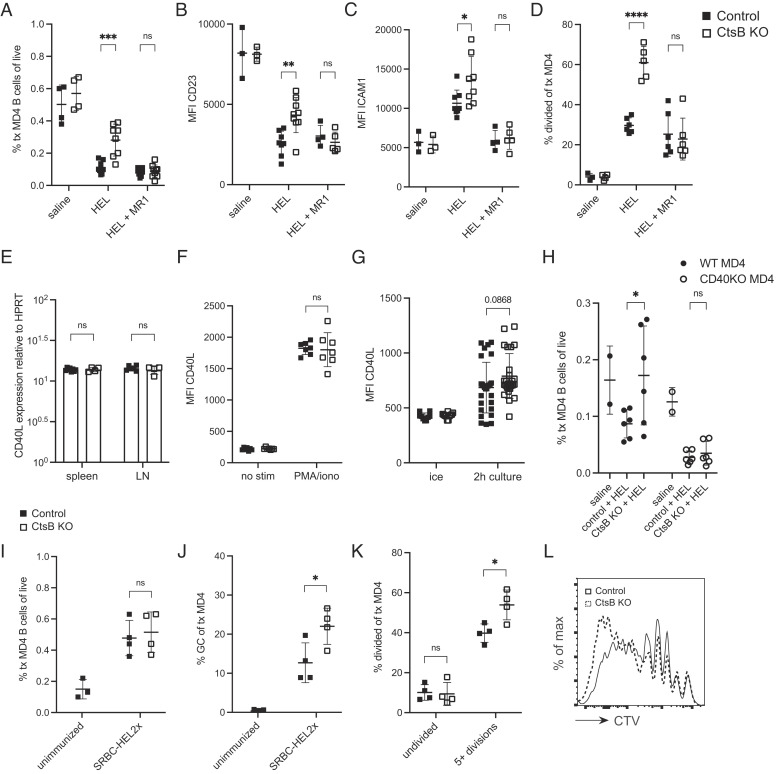
Enhanced HEL-Binding B Cell Persistence in Ctsb-deficient Hosts Depends on CD40L and CD40. (*A*) Frequencies of transferred MD4 B cells in spleens of control or Ctsb-deficient recipients 3 d after saline, HEL, or HEL with MR1 CD40L-blocking treatment. (*B* and *C*) MFI of CD23 (*B*) and ICAM1 (*C*) on transferred MD4 B cells 1.5 d after saline (n = 4 control, n = 4 KO), HEL (n = 8 control, n = 8 KO), or HEL with MR1 treatment (n = 9 control, n = 9 KO). (*D*) Percentage of divided transferred MD4 B cells 3 d after saline (n = 3 control, n = 4 KO), HEL (n = 6 control, n = 5 KO), or HEL with MR1 treatment (n = 6 control, n = 6 KO). (*E*) CD40L transcript abundance in naïve CD4^+^ T cells isolated from spleen and lymph node tissues harvested from control (n = 6) or Ctsb^−/−^ (n = 3) mice. CD40L and hypoxanthine phosphoribosyltransferase (HPRT) transcripts were quantitated by real-time PCR. Data show results from two separate experiments. (*F*) MFI of CD40L on purified CD4^+^ T cells from control (n = 7) or Ctsb-deficient (n = 8) mice incubated without (no stim) or with (PMA/iono) PMA and ionomycin for 2 h at 37 °C. (*G*) MFI of CD40L on purified CD4^+^ T cells from control (n = 27) or Ctsb-deficient (n = 26) mice kept on ice or incubated in a dilute culture for 2 h at 37 °C. (*H*) Frequencies of WT or CD40-deficient MD4 B cells in transferred splenocytes in spleens of control or Ctsb-deficient recipients 3 d after saline (n = 2 control, n = 2 KO) or HEL treatment (n = 6 control, n = 6 KO). (*I* and *J*) Frequencies of transferred MD4 B cells amongst total cells (*I*) and of MD4 B cells having a germinal center (GC) phenotype (*J*) in spleens of unimmunized mice (n = 3) or mice immunized with SRBC-HEL^2x^ (n = 4 control, n = 4 KO). (*K*) Frequencies of undivided MD4 B cells or MD4 B cells having undergone five or more divisions in spleens of control (n = 4) or Ctsb-deficient mice (n = 4) 5 d after SRBC-HEL^2x^ immunization. (*L*) Representative histogram plot of CTV labeling of transferred MD4 B cells in indicated recipients 5 d after SRBC-HEL^2x^ immunization. Each data point indicates an individual mouse and lines indicate means. Error bars represent SDs. *A*–*D* and *H* are representative of three experiments. *I*–*L* are representative of two experiments. Statistical significance for *A*–*K* was determined by unpaired *t* test. NS, not significant; *P < 0.05; **P < 0.01; ***P < 0.001; ****P < 0.0001.

Control and Ctsb-deficient mice were treated with CD40L-blocking antibody, and the fate of MD4 B cells after HEL treatment was examined. Blocking CD40L had a similar effect to CD4^+^ T cell ablation, overcoming the effect of Ctsb deficiency on HEL-binding B cell accumulation (Fig. 4*A*). This included a loss in CD23 and ICAM1 induction (Fig. 4 *B* and *C*) and a complete loss of increased cell division (Fig. 4*D*). We then examined CD40L transcript levels in sorted naïve CD4^+^ T cells and found they were abundant as expected (8) and were unaltered by Ctsb deficiency (Fig. 4*E*). Moreover, surface CD40L on PMA plus ionomycin-activated control and Ctsb-deficient CD4^+^ T cells was equivalent (Fig. 4*F*). CD40 engagement of CD40L causes loss of CD40L from the T cell surface (19–21); however, when purified naïve CD4^+^ T cells are incubated in a low-density culture and thus in the absence of CD40 exposure, CD40L becomes detectable on the cell surface (8). When control and Ctsb-deficient CD4^+^ T cells were incubated in this way, the Ctsb-deficient cells showed a trend for more surface display of CD40L, but this difference was not statistically significant (Fig. 4*G* and *SI Appendix*, Fig. S3*B*). Thus, under the conditions tested, Ctsb did not significantly alter CD40L expression. Finally, as a further test of the contribution of CD40L to the augmented survival of HEL-binding B cells in Ctsb-deficient hosts, we cotransferred WT and CD40^−/−^ MD4 B cells into control and Ctsb-deficient hosts and treated them with soluble HEL. The analysis 3 d later showed comparable deletion in both types of recipients (Fig. 4*H*). CD40-deficiency also prevented elevation of ICAM1 and IgM, though there was still some induction of CD23 (*SI Appendix*, Fig. S3 *C* and *D*). The expression of IgM was widely dispersed in this experiment (*SI Appendix*, Fig. S3*E*). CD40 abundance on WT MD4 B cells in control and Ctsb-deficient mice was similar (*SI Appendix*, Fig. S3*F*). Taken together, these data suggest that the effect of Ctsb on augmenting deletion of HEL-binding B cells involves a process that limits the CD40L-CD40 engagement that occurs when HEL-binding B cells encounter naïve CD4^+^ T cells in lymphoid tissues.

Although we did not observe an alteration in the polyclonal GC response to complex foreign or gut-associated antigens (*SI Appendix*, Fig. S1 *D* and *E*), our findings suggesting that noncognate T-dependent signals could be increased in the absence of Ctsb led us to perform a further experiment examining cognate T cell help. We transferred MD4 cells into control or Ctsb-deficient recipients and immunized the mice with HEL^2x^, a form of HEL with reduced affinity for the MD4 BCR (22), coupled to strongly immunogenic SRBCs. Upon analysis at 5 d, although total numbers of MD4 B cells were similar between recipients, we observed an increase in the fraction of MD4 cells that had a GC phenotype in the immunized Ctsb-deficient mice compared to control recipients (Fig. 4 *I* and *J*). Moreover, CTV dilution analysis showed that the MD4 cells had undergone more extensive cell division in the Ctsb-deficient recipients (Fig. 4 *K* and *L*). These data indicate that under some conditions, Ctsb can restrain the extent of T cell help in response to foreign antigen.

## Discussion

Here we find that the elimination of peripheral B cells experiencing chronic signal-1 is partially dependent on extrinsic Ctsb activity. Since more than one source of Ctsb (hematopoietic or nonhematopoietic) was sufficient, we speculate that Ctsb was acting in an extracellular manner to reduce the availability of a factor (or factors) that augments the survival and proliferation of chronically BCR-engaged B cells. Ctsb activity in promoting B cell elimination was lost when CD4^+^ T cells, CD40L, or CD40 was lacking. We suggest Ctsb acts in part by reducing signaling via the CD40L–CD40 axis during the encounter between autoantigen-binding B cells and naïve CD4^+^ T cells. The finding of increased GC B cells in Ctsb-deficient mice immunized with a model foreign antigen is in accord with a possible increase in CD40L–CD40 signaling. However, we cannot exclude the possibility that Ctsb is acting to restrain an independent pathway that cooperates with CD40L–CD40 or other T cell-derived signals. Overall, these findings identify a role for extracellular protease activity in establishing the extent of B cell tolerance to peripheral self-antigen and potentially the magnitude of the response to some foreign antigens.

A previous study showed that Ctsb could cleave CXCL13 in vitro and the truncated form was reported to be more potent in promoting B cell migration than the full-length form (16). Images were provided (without further quantification) suggesting that Ctsb-deficient mice have a loss of follicular structure and FDCs in lymph nodes. FDCs are maintained in lymphoid follicles via LTα1β2 signals provided by B cells in a partially CXCL13-dependent manner (23), making the observations in Ctsb-deficient mice internally consistent. In contrast to those observations, we failed to observe a defect in B cell follicular clustering in Ctsb-deficient mice, and the follicles contained intact FDC networks. The basis for this discrepancy between studies is unclear but might reflect different breeding histories of the mouse lines or differences in the microbiome between facilities. Although Ctsb was shown to be able to cleave CXCL13 in vitro, it was not shown that the enzyme has this activity in vivo (16). Since our anatomical findings suggest CXCL13 function does not depend on Ctsb, we have not examined whether CXCL13 is cleaved in vivo by Ctsb. Importantly, the influence of Ctsb on autoantigen-engaged B cell survival is unlikely to be via effects on CXCL13 as earlier studies have shown that CXCR5-deficient B cells that cannot respond to CXCL13 undergo an equivalent extent of deletion as WT B cells in the HEL-MD4 model (24).

Ctsb acts on a wide range of intracellular and extracellular substrates (9, 10). While our study does not identify the molecule(s) that Ctsb acts on to restrain autoreactive B cell survival, our findings in CD4^+^ T cell-deficient, CD40L-blocked, and CD40-deficient systems point to actions that influence the CD40L–CD40 pathway. This possibility is also supported by the finding of increased ICAM1 and CD23 expression in chronically antigen-engaged B cells in Ctsb-deficient hosts, as both these molecules are CD40-inducible (25, 26). The augmented proliferation in the case of B cells receiving chronic signal-1 and for MD4 B cells provided with cognate T cell help is also consistent with increased CD40 pathway activity. Our inability to detect significantly altered surface abundance of CD40L on CD4^+^ T cells or CD40 on B cells from Ctsb-deficient mice suggests Ctsb may affect this pathway in an indirect manner. However, it must be kept in mind that the activity of CD40L in vivo that augments autoreactive B cell survival likely reflects transient surface expression on naïve CD4^+^ T cells (8). Since we are not able to measure this low, transient surface CD40L, we cannot exclude that Ctsb, whether directly or indirectly, reduces the amount of CD40L available from naïve CD4^+^ T cells during interactions with chronically antigen-engaged B cells. In this regard, it is notable that a disintegrin and metalloprotease 10 (ADAM10) can promote reductions in surface CD40L (21). Work in cancer cell lines has indicated that Ctsb can promote the release of soluble ADAM10 (27). These observations raise the possibility that Ctsb, by increasing soluble ADAM10 in tissues, reduces the amount of CD40L available at interfaces between T cells and B cells, a possibility that merits exploration. Alternatively, the effect of Ctsb could be via a distinct pathway and this pathway can only show an influence on B cell survival and proliferation when the CD40L-CD40 pathway is intact; when this pathway is missing, the Ctsb-regulated pathway may be insufficient to have an influence. Future studies examining global gene expression changes in autoantigen-engaged B cells in control versus Ctsb-deficient recipients at different time points may help determine the signaling pathway(s) most influenced by Ctsb.

Our study provides an example of a protease acting to influence the extent of tolerance induction in lymphoid tissues. Although less extensively tested, our work suggests this activity may also restrain the amount of help provided during responses to some foreign antigens. Interestingly, a recent report found that deficiency in prolidase, a cytosolic metallopeptidase, causes spontaneous T cell activation and lupus-like autoimmunity (28). It will be of interest in the future to determine whether Ctsb deficiency predisposes to systemic autoimmune disease. Moreover, increases in protease activity are common at sites of inflammation and in tumors. It will be important in future studies to determine the extent to which immune checkpoints are reset in these sites due to proteolytic modulation of immune cell communication molecules.

## Materials and Methods

### Mice.

All mice were bred internally, and 6- to 20-wk-old mice of both sexes were used. Cathepsin-deficient [B6;129-*Ctsb^tm1Jde^*/J] mice were obtained from JAX and were backcrossed six times to C57BL/6J. CD40-deficient [B6.129P2-*Cd40^tm1Kik^*/J] mice were obtained from JAX. MD4 mice [C57BL/6-Tg(IghelMD4)4Ccg/J] and UBC-GFP [Tg(UBC-GFP)30Scha/J] mice were from an internal colony. ML5 mice [Tg(ML5sHEL)5Ccg] were obtained from Julie Zikherman, University of California, San Francisco, CA. CD45.1 B6 [B6.SJL-PtprcaPepcb/BoyCrCrl] mice used for chimera recipients and cell transfer experiments were bred internally from founders ordered from JAX. In most experiments, littermates were used as controls and experimental animals were co-caged in groups of two to six whenever possible. All mice were analyzed between 8 and 20 wk of age. Animals were housed in a specific pathogen-free environment in the Laboratory Animal Research Center at UCSF, and all experiments conformed to ethical principles and guidelines approved by the UCSF Institutional Animal Care and Use Committee.

### Cathepsin B Activity Assay.

Spleens from control and Ctsb^−/−^ mice were mashed through a 70-µm cell strainer in 700μL of PBS. Resulting cell suspensions were centrifuged at 1,500 rpm at 4 °C to pellet cells. The interstitial fluid-containing supernatant (or “mashate”) was collected, diluted 1:10, and Ctsb activity was measured using a fluorometric Ctsb activity assay (Abcam).

### Adoptive Transfer of MD4 B Cells.

Spleens and lymph nodes from CD45.1 MD4 mice were macerated and resulting cell suspensions were filtered through a 70-μm mesh into PBS supplemented with 2% FCS and 1mM EDTA. Counting beads were used for the enumeration of cells, and frequency of MD4 B cells was determined by staining of IgMa-positive B cells on the flow cytometer. Cells were labeled with Cell Trace Violet (CTV) (Invitrogen) and 5 to 10 × 10^6^ MD4 B cells were injected intravenously into recipient mice. For positioning of HEL-binding B cells, spleens and lymph nodes from CD45.1 MD4 GFP mice were prepared as above. MD4 B cells were enriched by negative selection using biotinylated antibodies against CD43, CD4, CD8, TCR*β*, CD11c, and Ter119, followed by streptavidin-conjugated beads (EasySep Streptavidin RapidSpheres) to greater than 95% purity. Counting beads were used for the enumeration of cells, and 5 to 10 × 10^6^ MD4 B cells were injected intravenously into recipient mice. For CD40-deficient MD4 experiments, CD45.1 WT MD4 and CD45.2 CD40^−/−^ MD4 spleens and lymph nodes were prepared as above. WT and CD40^−/−^ MD4 splenocytes were mixed such that the MD4 B cells reached a 1:1 ratio. Cells were labeled with CTV, and 10 to 15 × 10^6^ MD4 B cells were injected intravenously into recipient mice.

### HEL and Antibody Treatments.

Recipient mice were injected intravenously with 1 mg hen egg lysozyme (HEL) (Sigma) 1 d after adoptive transfer of MD4 B cells. For CD4^+^ T cell depletion experiments, 250 μg anti-mouse CD4 GK1.5 monoclonal antibody (BioXCell) was injected intravenously 2 to 3 h before HEL treatment on d0 and on d1.5. For CD40L blocking experiments, 250 μg anti-mouse CD40L MR1 monoclonal antibody (BioXCell) was injected intravenously on d0 and d1.5.

### HEL Serum Measurements.

Serum was collected from control or Ctsb^−/−^ mice 3 d after 1 mg HEL treatment or from control ML5^+^ or Ctsb^−/−^ ML5^+^ mice. MD4 B cells were plated in a 96-well plate at 5 × 10^6^ cells per mL in PBS supplemented with 2% FCS and incubated with sera at 1:10 and 1:50 dilutions for 20 min on ice. Cells were washed three times and HEL occupancy was measured by staining with HyHEL9–PE-Cy5.5 (in house).

### Bone Marrow Chimeras.

CD45.1 B6 or Ctsb^−/−^ mice were lethally irradiated with 1,100 rads gamma irradiation (split dose separated by 3 h) and then i.v. injected with relevant BM cells under isoflurane anesthesia. Chimeras were used as recipients for adoptive transfer experiments after 8 to 10 wks of reconstitution.

### Immunizations.

Recipient mice were immunized intravenously with 2 × 10^8^ SRBC (Colorado Serum Company) in a volume of 200 μL. Spleens were harvested on day 5. For SRBC-HEL^2×^ experiments, 1 × 10^5^ MD4 B cells were CTV-labeled and transferred into recipient mice. The following day, mice were immunized intravenously with 2 × 10^8^ SRBC-HEL^2×^, and spleens were harvested on day 5 post-immunization. Conjugation of SRBC-HEL^2×^ was done as previously described (29). Briefly, SRBCs were first washed with PBS three times, mixed with 10 μg/mL HEL^2×^ (Gift of R. Brink), crosslinked with EDCI (1-ethyl-3-(3-dimethylaminopropyl) carbodiimide) (Sigma-Aldrich) for 30 min, and washed three times to remove the free HEL.

### Immunofluorescence.

Lymph nodes and spleens were embedded in optimal cutting temperature compound. Cryosections of 7 μm were dried for 1 h, fixed in acetone for 10 min, and dried for 1 h at room temperature. Slides were rehydrated in PBS containing 0.1% fatty acid-free bovine serum albumin (BSA) for 10 min. A solution consisting of 1% normal mouse serum (NMS), 1% normal donkey serum (NDS), 1:100 AF647-conjugated anti-mouse CD21/35 (BD Bioscience, catalog no. 123424) and 1:300 goat anti-mouse IgD (Cedarlane Laboratories, GAM/IGD(FC)/7S) was used to label FDCs and endogenous naïve B cells, respectively. This solution was incubated with the slides overnight at 4 °C. The slides were then washed in PBS and stained with AMCA-conjugated donkey anti-goat IgG (Jackson Immunoresearch, 705-156-147) at room temperature, and images were captured with a Zeiss AxioObserver Z1 inverted microscope.

To track the positioning of GFP MD4 B cells, spleens were fixed in 4% paraformaldehyde (PFA) for 2 h at 4 °C, washed with phosphate-buffered saline (PBS), submerged in 30% sucrose overnight, and embedded in optimal cutting temperature compound. Cryosections of 7 μm were dried for 1 h at room temperature and rehydrated in PBS containing 0.1% fatty acid-free bovine serum albumin (BSA) for 10 min. A solution consisting of 1% NMS and NDS, 1:100 AF488-conjugated rabbit anti-GFP (Invitrogen, catalog no. A21311), 1:100 AF647-conjugated anti-mouse CD21/35, 1:100 biotin-conjugated anti-mouse CD4 (BioLegend, catalog no. 100404), and 1:300 goat anti-mouse IgD was used to label MD4 B cells, FDCs, endogenous CD4 T cells, and endogenous naïve B cells, respectively. These solutions were incubated with the slides overnight at 4 °C. The slides were then washed in PBS and stained with Cy3-conjugated streptavidin (Jackson Immunoresearch, catalog no. 016-160-084) and AMCA-conjugated donkey anti-goat IgG for 1 h at room temperature, and images were captured with a Zeiss AxioObserver Z1 inverted microscope.

### Quantification of IF Images.

Images of immunofluorescent stains of GFP MD4 B cell positioning were quantified using ImageJ (version 1.53). All images were captured at the same magnification using a Zeiss AxioObserver Z1 inverted microscope. Images were loaded into ImageJ and the freehand line tool was used to outline the T-B interface of a follicle. The total number of GFP MD4 B cells 20 μm on either side of the T-B interface was quantified. The number of GFP MD4 B cells inside the follicle was also quantified, and the proportion of GFP MD4 B cells at the T-B interface to GFP MD4 B cells in the follicle was calculated.

### Cell Culture.

CD4^+^ T cells from control and Ctsb^−/−^ lymph nodes were enriched by negative selection using biotinylated antibodies against B220, CD8, CD11c, and Ter119, followed by streptavidin-conjugated beads to greater than 95% purity. Cells were plated in a 96-well plate at 5 × 10^6^ cells per mL in RPMI medium 1640 plus 10% FCS at 37 °C and rested or stimulated for 2 h with 10 ng/mL PMA and 1 μg/mL ionomycin. To facilitate the detection of surface-exposed CD40L, the cells were incubated in the presence of 1 mg/mL anti-CD40L antibody as previously described (30, 31). To mimic CD40 deficiency, we plated CD4^+^ T cells from control and Ctsb^−/−^ lymph nodes in a dilute culture of 0.5 mL at 5 × 10^5^ cells per mL in 24-well plates and kept on ice or incubated at 37 °C for 2 h.

### Real-Time PCR Analysis.

B6 and Ctsb^−/−^ spleens and lymph nodes were harvested and CD4^+^ T cells were enriched by negative selection using biotinylated antibodies against CD19, B220, CD8, CD11c, and Ter119, followed by streptavidin-conjugated beads to greater than 95% purity. Cell pellets were snap-frozen in liquid nitrogen, and RNA was prepared by using an RNeasy kit (Qiagen). Equivalent amounts of cDNA were used in quantitative PCR on an ABI 7300 sequence detection instrument (Applied Biosystems) by using primer sets with SYBR Green (Bio-Rad). Primer pairs were as follows (forward, F; reverse, R): hypoxanthine phosphoribosyltransferase (HPRT) F, AGGTTGCAAGCTTGCTGGT, and HPRT R, TGAAGTACTCATTATAGTCAAGGGCA; CD40 ligand F, GTGAGGAGATGAGAAGGCAA, and CD40 ligand R, CACTGTAGAACGGATGCTGC.

### Flow Cytometry.

Cells were stained for 20 min on ice in MACS buffer (2% FCS in PBS with 1 mM EDTA) at 0.5 to 1 × 10^6^ cells per well in 96-well round-bottom plates unless otherwise specified. The following monoclonal antibodies were used: B220–BV785 (BioLegend), ICAM1–biotin (BD), followed by streptavidin–BV711 (Fisher), CD45.2–PerCP-Cy5.5 (Tonbo), IgMa–FITC (Fisher), CD40–PE (Fisher), CD23–PE-Cy7 (BioLegend), CD45.1–BV605 (BioLegend), CD40L–PE (BioLegend), and CD40L–biotin (eBioscience), followed by streptavidin–A647 (Fisher). Dead cells were excluded using Fixable Viability Dye eFluor780 (eBioscience no. 65-0865-18). All samples were run on a BD LSRII or BD FACSymphony A1 at 5,000 to 10,000 events per second. Flow cytometry data were analyzed using FlowJo (v10.8.1).

### BAFF Staining.

Staining for BAFF occupancy of BAFFR on B cells was done as previously described (5). In brief, after FcR blocking, spleen cells were incubated with rabbit anti-mouse BAFF-ectodomain serum at a 1:1,000 dilution, followed by 1:100 goat anti-rabbit–biotin with 2% NMS, normal rat serum (NRS), and normal goat serum (NGS), followed by streptavidin–A647 and antibodies to other markers. As a positive control, cells were incubated with recombinant BAFF prior to the antibody staining.

## Statistical Analyses.

Data were analyzed using unpaired Student’s *t* test, and ordinary one-way ANOVA using Tukey’s multiple comparisons test was performed when comparing one variable across multiple groups. Prism version 9 (GraphPad Software) was used for all statistical analyses and to generate plots. Each experiment was repeated at least three times, unless otherwise indicated in the figure legends. In summary graphs, points indicate individual samples and horizontal lines are means. All error bars represent SDs.

## Supplementary Material

Appendix 01 (PDF)

## Data Availability

All study data are included in this article and/or *SI Appendix*.
