# Supplementary material for: B cell peripheral tolerance is promoted by cathepsin B protease
Source: Proc Natl Acad Sci U S A. 2023 Apr 11;120(16):e2300099120. doi: 10.1073/pnas.2300099120 (PMC10120085; doi:10.1073/pnas.2300099120)
Supplement: Supplementary file 1 — Appendix 01 (PDF) [file pnas.2300099120.sapp.pdf]

PNAS

Supporting Information for
B cell peripheral tolerance is promoted by cathepsin B protease

Marissa Y. Chou, Dan Liu, Jinping An, Ying Xu, Jason G. Cyster

Jason G. Cyster
Email: jason.cyster@ucsf.edu

This PDF file includes:

Figures S1 to S3

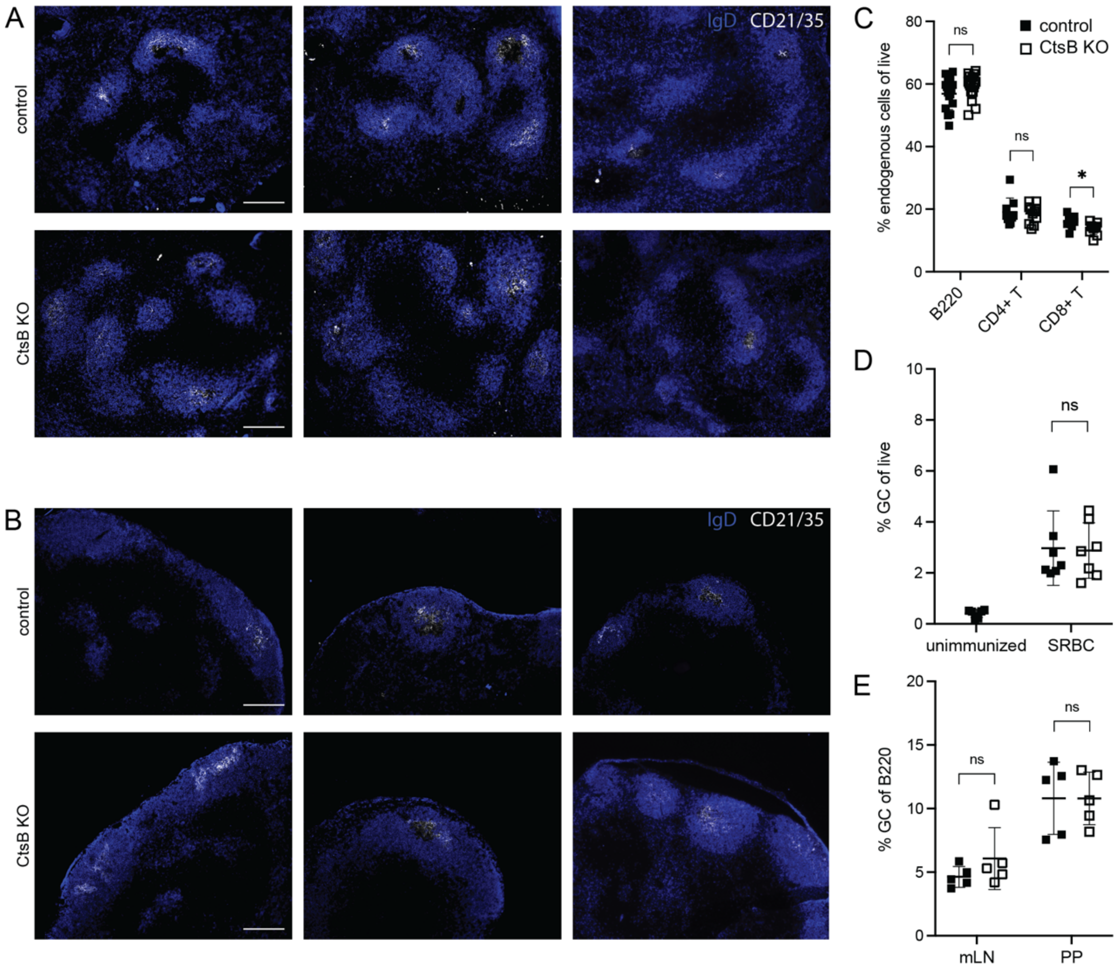

Supplemental Figure 1

Fig. S1. (A, B) Immunofluorescence for B cell follicles (IgD, blue) and FDCs (CD21/35, white) in spleen (A) and peripheral lymph nodes (B) from control (Ctsb$^{+/+}$ or Ctsb$^{+/-}$, top row) or Ctsb$^{-/-}$ (bottom row) mice. Scale bar, 200 μm. Three example images are shown and are representative of multiple cross sections from at least three mice of each type. (C) Frequencies of B, CD4^{+} T, and CD8^{+} T cells in spleens of control or Ctsb-deficient mice (N ≥ 9 mice per genotype). (D) Frequencies of germinal center (GC) B cells in spleens of unimmunized mice (n = 6), or control (n = 7) or Ctsb-deficient (n = 7) mice 5 days after sheep red blood cell (SRBC) immunization. (E) Frequencies of GC B cells in mesenteric lymph nodes or Peyer's patches of control (n = 5) or Ctsb-deficient (n = 5) mice at homeostasis. Each data point indicates an individual mouse and lines indicate means. Error bars represent SDs. D is representative of three experiments. Statistical significance for C–E was determined by unpaired t test. NS, not significant; *$P < 0.05$.

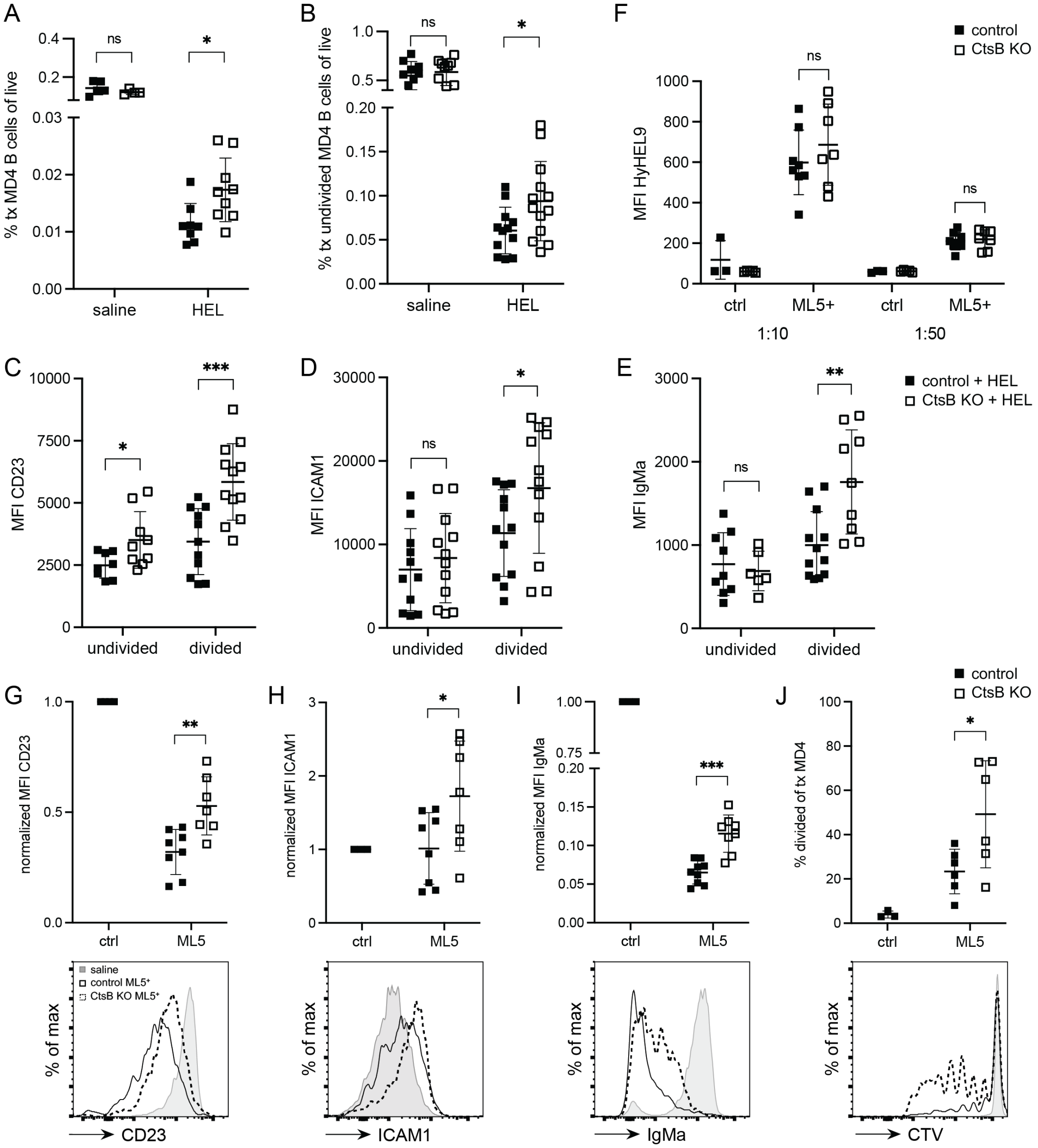

Supplemental Figure 2

Fig. S2. (A) Frequencies of transferred MD4 B cells in lymph nodes of control or Ctsb-deficient recipients 3 days after saline (n = 5 control, n = 4 KO mice) or HEL treatment (n = 8 control, n = 9 KO). (B) Frequencies of undivided (CTV-high) transferred MD4 B cells in spleens of control or Ctsb-deficient recipients 3 days after saline (n = 10 control, n = 10 KO) or HEL treatment (n = 12 control, n = 13 KO). (C–E) MFI of CD23 (C), ICAM1 (D), and IgMa (E) on undivided and divided transferred MD4 B cells in control or Ctsb-deficient recipients 3 days after HEL treatment (N ≥ 6 mice per genotype). (F) MFI of HyHEL9 on MD4 B cells incubated with 1:10 or 1:50 dilutions of sera from control ML5⁻ (n = 3), Ctsb-deficient ML5⁻ (n = 5), control ML5⁺ (n = 8), or Ctsb-deficient ML5⁺ (n = 7) mice. (G–I) Normalized MFI (top) and representative histogram plot (bottom) of CD23 (G), ICAM1 (H), and IgMa (I) on transferred MD4 B cells in control ML5⁺ (n = 8) or Ctsb-deficient ML5⁺ (n = 7) mice 3 days after MD4 B cell adoptive transfer. Control ML5⁻ (n = 4) mice

used as deletion control. (J) Percentage of divided transferred MD4 B cells (top) or representative histogram plot of CTV (bottom) in control $ML5^+$ (n = 6) or Ctsb-deficient $ML5^+$ (n = 6) mice 3 days after MD4 B cell adoptive transfer. Control $ML5^-$ (n = 3) mice used as deletion control. Each data point indicates an individual mouse and lines indicate means. Error bars represent SDs. A and C–J are representative of three experiments. Statistical significance for A–J was determined by unpaired t test. NS, not significant; *P < 0.05; **P < 0.01.

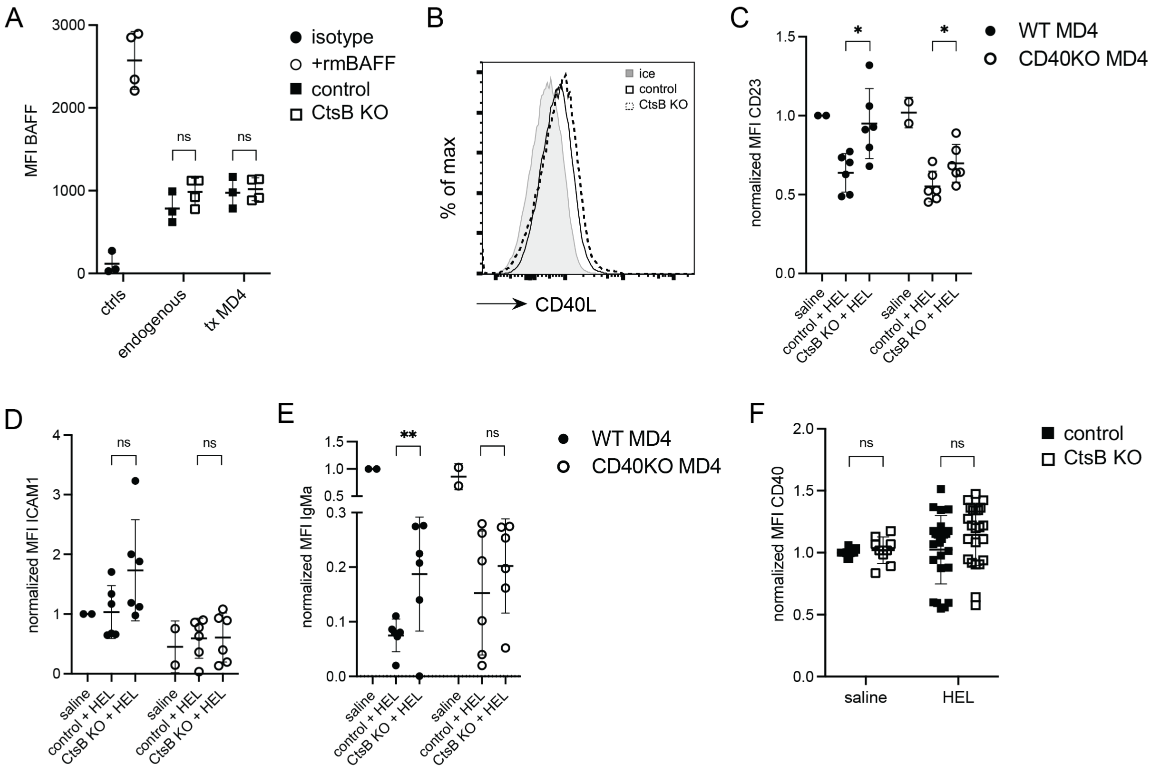

Supplemental Figure 3

Fig. S3. (A) MFI of anti-BAFF staining on endogenous B cells and transferred MD4 B cells at day 3 in control (n = 3) or Ctsb-deficient (n = 3) mice. Control column (ctrls) indicates staining with control antibody (isotype) or pre-incubation with 10 µg/mL recombinant mouse BAFF (+rmBAFF) prior to anti-BAFF staining. (B) Representative histogram plot of CD40L on purified CD4$^+$ T cells kept on ice or incubated in a dilute culture for 2 h at 37°C. (C–E) Normalized MFI of CD23 (C), ICAM1 (D), and IgMa (E) on transferred WT or CD40-deficient MD4 B cells 3 days after saline (n = 2 control, n = 2 KO) or HEL treatment (n = 6 control, n = 6 KO). (F) Normalized MFI of CD40 on transferred MD4 B cells 3 days after saline (n = 11 control, n = 9 KO) or HEL treatment (n = 24 control, n = 23 KO). Each data point indicates an individual mouse and lines indicate means. Error bars represent SDs. A and C–E are representative of three experiments. Statistical significance for A and C–F was determined by unpaired t test. NS, not significant; *P < 0.05; **P < 0.01.
